# Voltage-Dependent Anion-Selective Channels and Other Mitochondrial Membrane Proteins Form Diverse Complexes in Beetroots Subjected to Flood-Induced Programmed Cell Death

**DOI:** 10.3389/fpls.2021.714847

**Published:** 2021-09-08

**Authors:** Karla J. Rojas-Méndez, Lino Sánchez Segura, Alicia Chagolla, Bárbara Lino, Luis E. González de la Vara

**Affiliations:** ^1^Laboratorio de Bioenergética y Biomembranas, Departamento de Biotecnología y Bioquímica, Unidad Irapuato, Centro de Investigación y de Estudios Avanzados del IPN, Irapuato, Mexico; ^2^Laboratorio de Microscopía, Departamento de Ingeniería Genética, Unidad Irapuato, Centro de Investigación y de Estudios Avanzados del IPN, Irapuato, Mexico; ^3^Laboratorio de Proteómica, Departamento de Biotecnología y Bioquímica, Unidad Irapuato, Centro de Investigación y de Estudios Avanzados del IPN, Irapuato, Mexico

**Keywords:** beetroot (*Beta vulgaris*), cytochrome c release, flooding stress, mitochondrial membrane protein complexes, programmed cell death (PCD), voltage-dependent anion-selective channels (VDAC)

## Abstract

In plants, programmed cell death (PCD) is involved in both the development and the response to biotic and abiotic aggressions. In early stages of PCD, mitochondrial membranes are made permeable by the formation of permeability transition pores, whose protein composition is debated. Cytochrome c (cyt c) is then released from mitochondria, inducing the degradation of chromatin characteristic of PCD. Since flooding stress can produce PCD in several plant species, the first goal of this study was to know if flooding stress could be used to induce PCD in *Beta vulgaris* roots. To do this, 2-month-old beet plants were flood-stressed from 1 to 5 days, and the alterations indicating PCD in stressed beetroot cells were observed with a confocal fluorescence microscope. As expected, nuclei were deformed, and chromatin was condensed and fragmented in flooded beetroots. In addition, cyt c was released from mitochondria. After assessing that flood stress induced PCD in beetroots, the composition of mitochondrial protein complexes was observed in control and flood-stressed beetroots. Protein complexes from isolated mitochondria were separated by native gel electrophoresis, and their proteins were identified by mass spectrometry. The spectra count of three isoforms of voltage-dependent anion-selective channels (VDACs) increased after 1 day of flooding. In addition, the size of the complexes formed by VDAC was higher in flood-stressed beetroots for 1 day (∼200 kDa) compared with non-stressed ones (∼100 kDa). Other proteins, such as chaperonin CPN60-2, also formed complexes with different masses in control and flood-stressed beetroots. Finally, possible interactions of VDAC with other proteins were found performing a cluster analysis. These results indicate that mitochondrial protein complexes formed by VDAC could be involved in the process of PCD in flood-stressed beetroots. Data are available *via* ProteomeXchange with identifier PXD027781.

## Introduction

Programmed cell death (PCD) has been reported to be essential for the development of plants (Greenberg, 1996). This process is also implicated in senescence and defense against biotic and abiotic aggressions (Lam et al., 2001). Mitochondria play a dominant role in stress-induced cell death since this phenomenon is initiated when mitochondria perceive death signals *via* elevated cytoplasmic Ca^2+^ and reactive oxygen species (ROS) (Petrov et al., 2015). Then, mitochondrial membranes become permeable through the opening of mitochondrial permeability transition pores (MPTPs). The ensuing solute flow through MPTP causes mitochondrial swelling and disruption of the outer mitochondrial membrane (OMM). This allows the release of proteins, such as cytochrome c (cyt c), from the mitochondrial intermembrane space to the cytoplasm (Van Aken and Van Breusegem, 2015). There, cyt c could initiate a series of reactions ending in massive proteolysis, nucleolysis, and cell disintegration (Dickman et al., 2017).

Among the mitochondrial proteins involved in PCD are voltage-dependent anion-selective channels (VDACs). These proteins are most abundant in the OMM (Takahashi and Tateda, 2013). VDAC is involved in PCD in two ways: first, as the binding site of pro- and antiapoptotic cytoplasmic proteins, and second, as a proposed component of MPTP. Among the antiapoptotic VDAC-binding proteins found in both animals and plants is hexokinase (HK). This enzyme, associated with VDAC, not only catalyzes the phosphorylation of glucose using preferentially the ATP produced by respiring mitochondria (Pedersen, 2008; Alcántar-Aguirre et al., 2013) but also inhibits PCD (Godbole et al., 2013, reviewed in Rodríguez Saavedra et al., 2021). In contrast, it has been proposed that VDAC associates with some mitochondrial inner membrane proteins, such as the adenine nucleotide and/or the phosphate translocators, to form MPTP (Takahashi and Tateda, 2013; Hurst et al., 2017). VDAC oligomerization could also form protein channels through which PCD-initiating proteins could leak out the mitochondria into the cytoplasm (Shoshan-Barmatz et al., 2015, 2018).

Several abiotic stress conditions, such as heat (Balk et al., 1999; Vacca et al., 2004), cold (Koukalová et al., 1997), salt (Wang et al., 2010), or UV radiation (Danon and Gallois, 1998), induced PCD in different plant organs and species (reviewed in Reape and McCabe, 2008; Lord and Gunawardena, 2012). Flooding, one of the most severe types of abiotic stress that causes significant economic losses (Hirabayashi et al., 2013), can also produce PCD in plant cells (Chen et al., 2014; Qi et al., 2018). The immediate consequence of flooding is the decrease in the concentration of available oxygen in the soil. This hypoxia causes extensive alterations in the mitochondrial morphology after few hours of flooding that could lead to either adaptive metabolic changes or irreversible destruction of cell organelles (Vartapetian et al., 2003; Voesenek and Bailey-Serres, 2015). In the roots of some plant species, the death of specific cells causes the formation of aerenchyma, an air-filled tissue that enhances the ventilation of root cells to relieve the hypoxia caused by flooding (Evans, 2003; Voesenek and Bailey-Serres, 2015; Ni et al., 2019). Large changes in the amounts and activities of several mitochondrial proteins have also been observed in flood-stressed plants (Komatsu et al., 2011; Kamal and Komatsu, 2015).

Since *Beta vulgaris* roots are reported to be sensitive to hypoxia (Petraglia and Poole, 1980), it is conceivable that flooding could be an easy and adequate method to induce PCD in bulky roots similar to them. Then, after observing changes in the cell and nuclear morphology associated with cell death and the leakage of cyt c from mitochondria in flood-treated beetroots, it was concluded that flood stress could cause PCD in them. Mitochondria from non-stressed and flood-stressed beetroots were then isolated, and their protein complexes were separated by Blue Native gel electrophoresis (Wittig et al., 2006). The proteins in these complexes were identified, and their abundances were estimated, by mass spectrometry. The possible interactions of VDAC with themselves and with other proteins were observed. The results of these observations suggested that VDAC proteins could be involved in PCD induced by flood stress in plants.

## Materials and Methods

### Biological Material and Flooding Treatment

Beetroots were grown in a greenhouse under natural conditions as described by Lino et al. (2016). Two months after the seedlings were transplanted to pots, they were subjected to flooding stress by submerging the plant-containing pots in water-filled buckets until the water level reached 3 cm above the soil surface. Plants were subjected to this treatment for a period of 1–5 days. Control plants were irrigated as needed, allowing adequate drainage of the water.

### Preparation, Staining, and Microscopy

Beetroots were cut into small pieces (∼1 cm^3^), fixed with a 4% formaldehyde solution for 24 h, and dehydrated with a sucrose solution series (from 10 to 40%) for 24 h in each solution. The samples were sliced manually in thick sections (∼1 mm) and washed with distilled water for 10 min. The sections were stained incubating them in a dilute solution (1:100 from stock) of SynaptoRed^TM^ C2 (Sigma-Aldrich, St. Louis, MO, United States) for 1 h at 4°C in darkness. The sections were then washed with distilled water for 15 min, covered with a dilute solution (1:1,000 from a stock) of 4′,6-Diamidino-2-phenylindole dihydrochloride (DAPI; Sigma-Aldrich, St. Louis, MO, United States), and incubated for 10 min at 4°C in darkness. These sections were mounted on glass slides, covered with high-performance Zeiss cover glasses, and observed in a multiphoton microscope system (LSM 880–NLO, Zeiss, Oberkochen, Germany) equipped with a Ti:Sapphire laser (Chameleon Vision II, COHERENT, Santa Clara, CA, United States; tuning range: 690–1,060 nm). The samples were observed and analyzed with Immersion Objective 40×/1.3 Oil DIC, NA ∞/0.17, Zeiss Plan NEOFLUAR. DAPI was excited with the chameleon laser tuned at 750 nm with 0.7% of power and detected at 403–492 nm. SynaptoRed^TM^ C2 was excited with an Argon laser at 514 nm, and the emission from 625 to 750 nm was recorded. All micrographs were captured in CZI format at 1,131 × 1,131 pixels and RGB color.

To estimate the size and shape of nuclei, 60 micrographs were captured at 1.5X–1.5Y zoom, equivalent to 8,100 μm^2^ of the scanned area. The morphometric analysis of nuclei was carried out with a “cell counter notice” plugin, and the morphometric description was performed by “shape_descriptor1u” in ImageJ version 1.49p software (National Institutes of Health) as described by Filippi-Chiela et al. (2012).

### Preparation of Mitochondria

Mitochondria were prepared as described by Alcántar-Aguirre et al. (2013) with modifications. In brief, 120 g of beetroots were shredded and soaked in 200 ml of a medium containing 300 mM sucrose, 4 mM dithiothreitol (DTT), 3 mM ethylenediaminetetraacetic acid (EDTA), 0.1% bovine serum albumin (BSA), 0.5% w/v polyvinylpolypyrrolidone (insoluble PVP), 0.2 mM phenylmethylsulfonyl fluoride (PMSF), and 70 mM Tris–HCl (pH 8.0). This mixture was homogenized for 30 s with a Braun hand blender. The homogenate was filtered through a fine mesh and centrifuged at 6,000 × *g* for 20 min. The supernatant was collected and centrifuged at 21,000 × *g* for 20 min. The pellets were suspended in a washing medium (0.3 M mannitol, 10 mM 3-morpholinopropane-1-sulfonic acid, potassium salt buffer pH 7.4, and 1 mM EDTA) and centrifuged at 3,000 × *g* for 20 min; the supernatant was then centrifuged at 21,000 × *g* for 20 min. The pellet obtained was resuspended in a washing medium, mixed with Percoll to get a concentration of 28% (w/v), and centrifuged at 40,000 × *g* for 1 h at 4°C. Mitochondria were collected in the light-brown broad bands near the top of the Percoll gradient formed during the centrifugation. This was mixed with 15 times its volume of washing medium and centrifuged at 12,000 × *g* for 15 min. The pellets were suspended in a small volume of 25 mM Tris–MES (pH 7.5), 2 mM EDTA, 1 mM DTT, and 45% (v/v) glycerol and kept at –70°C until use.

### Immunodetection of cyt c and VDAC

To detect cyt c, samples from either mitochondria or cytoplasm (i.e., the supernatant of the first 21,000 × *g* centrifugation) were serially diluted. A total of 5 μl of each dilution were applied to a nitrocellulose membrane, dried, and subjected to immunodetection as described by González de la Vara and Lino Alfaro (2009). The primary antibody used was an anti-*Arabidopsis* cyt c antiserum (Agrisera, Vännäs, Sweden). Blots were revealed colorimetrically with 5-bromo-4-chloro-3-indolyl phosphate and nitro blue tetrazolium, as reported by González de la Vara and Medina (1990). In the same way, VDAC relative abundance was estimated in prepared mitochondria using anti-*Arabidopsis* VDAC rabbit antibodies (Agrisera, Vännäs, Sweden).

### Oxidative Phosphorylation and HK Activity

The ATP production by respiring mitochondria from control and flood-stressed beetroots was measured in a 0.5-ml enzymatic coupled assay system containing 0.3 M mannitol, 3 mM MgSO_4_, 10 mM NaCl, 1 mM glucose, 0.3 mM β-NADP, 0.07 U ml^–1^ yeast HK, 0.5 U ml^–1^ of *Saccharomyces cerevisiae* glucose-6P-dehydrogenase (in 50 mM Tris–HCl buffer, pH 7.4), 0.1% (w/v) fatty-acid-free BSA, 10 mM Na phosphate, 50 mM Tris–HCl buffer (pH 7.4), 0.15 mM ADP, and recently prepared mitochondria. Reactions were started by adding 10 mM potassium succinate. NADP reduction, indicating ATP production, was measured by recording the absorbance at 340 nm every 15 s in a Beckman DU-640 spectrophotometer (Brea, CA, United States).

The HK activity was determined in a medium containing 50 mM Tris–HCl buffer (pH 7.4), 6 mM MgCl_2_, 1 mM glucose, 1 mM ATP, 0.3 mM β-NADP, and 1 U ml^–1^ glucose-6P-dehydrogenase (NADP-dependent, from *S. cerevisiae*). The reaction was started by adding freshly prepared mitochondria. The activity was determined by measuring the NADPH formation through its absorption at 340 nm, using an extinction coefficient of 6.22 mM^–1^cm^–1^.

### Native Gel Electrophoresis

Shredded beetroots were treated with 0.4% w/v formaldehyde (prepared as described in the study by Pertl-Obermeyer et al., 2014) in a 20 mM sodium phosphate buffer (pH 7.5) for 20 min to cross-link proteins. Later, 125 mM glycine was added to stop this reaction. Mitochondria were then prepared from these formaldehyde-treated roots as described. However, when this cross-linking step was omitted, very similar results were obtained (data not shown).

Mitochondria were treated with the detergent dodecylmaltoside (DDM) at a detergent/protein ratio of 2. The separation of membrane protein complexes was performed by Blue Native Polyacrylamide Gel Electrophoresis (BN-PAGE), in 4–13% polyacrylamide gradient gels, as described by Wittig et al. (2006). Proteins in gels were fixed with 50% (v/v) methanol and 10% (v/v) acetic acid in water and stained with Coomassie Blue (Schägger and von Jagow, 1987). Mitochondrial complexes in a membrane preparation from the chicken heart were used as molecular mass markers (Wittig et al., 2010).

### Mass Spectrometry and Data Processing

Lanes in BN-PAGE gels were sliced as indicated in Figure 6. Proteins in these slices were processed for mass spectrometry as described (Lino et al., 2006), following the method of Shevchenko et al. (1996).

The peptides obtained were analyzed in a linear ion trap LTQ Velos mass spectrometer (Thermo Fisher Scientific, Bremen, Germany) online with a nanoACQUITY (Waters Corp., Milford, MA, United States) liquid chromatography system, with the conditions described by Lino et al. (2016).

The data were analyzed using a *Trans-*Proteome Pipeline platform (Deutsch et al., 2010), and the results were validated with the PeptideProphet and ProteinProphet software included in this platform. The fragmentation spectra were compared with theoretical ones from a *B. vulgaris* database (Dohm et al., 2014) obtained from NCBI. Proteins with a ProteinProphet probability >0.9999 and, at least, five unique peptides were considered as positively identified. To group the proteins according to their distribution among complexes of different masses, the software NOVA (Giese et al., 2015) was used, choosing the normalization and Pearson’s correlation parameters.

## Results

### Flooding Produced Morphological Changes Suggestive of PCD in Beetroots

**FIGURE 1:**
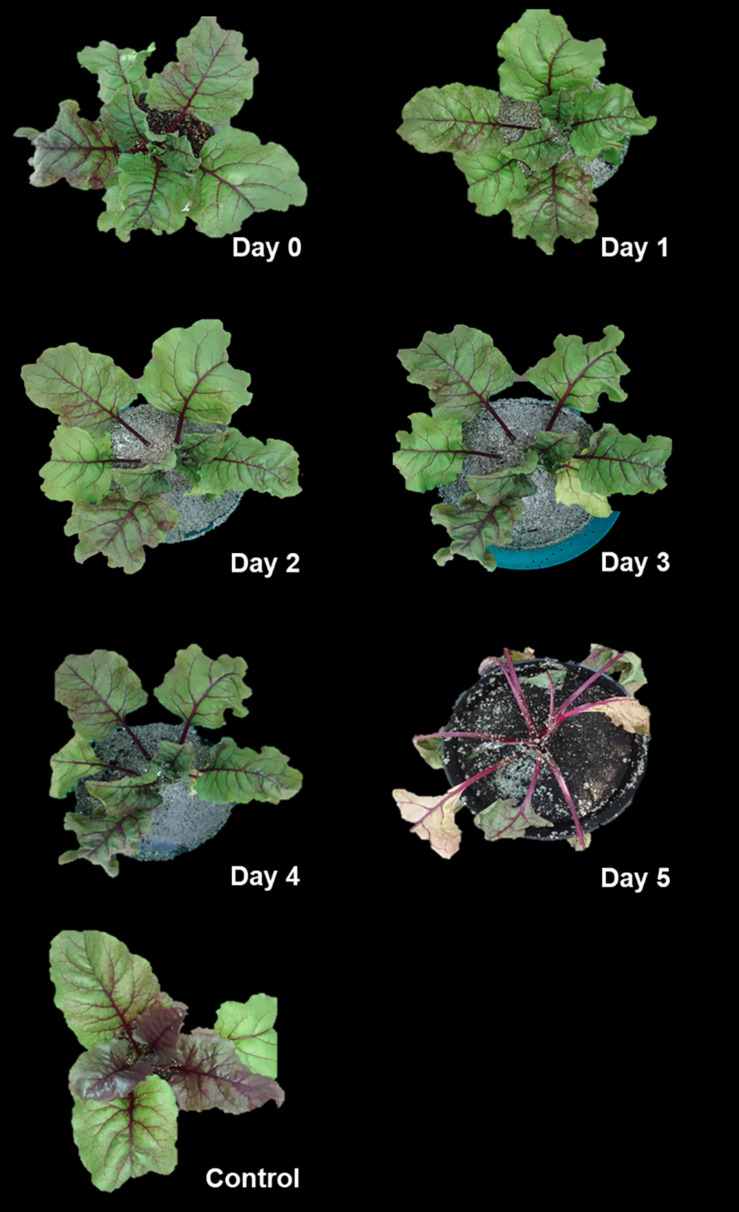
Flooding produces wilting in *Beta vulgaris* plants. Two-month-old plants were flooded for periods of 1–5 days, as indicated. A control plant, which was irrigated in well-drained soil, is also shown.

When 2-month-old *B. vulgaris* plants were flooded, damages in leaves were apparent in the following few days. They presented several symptoms such as wilting, chlorosis, and vein clearing, especially after 5 days of flooding (Figure 1).

**FIGURE 2:**
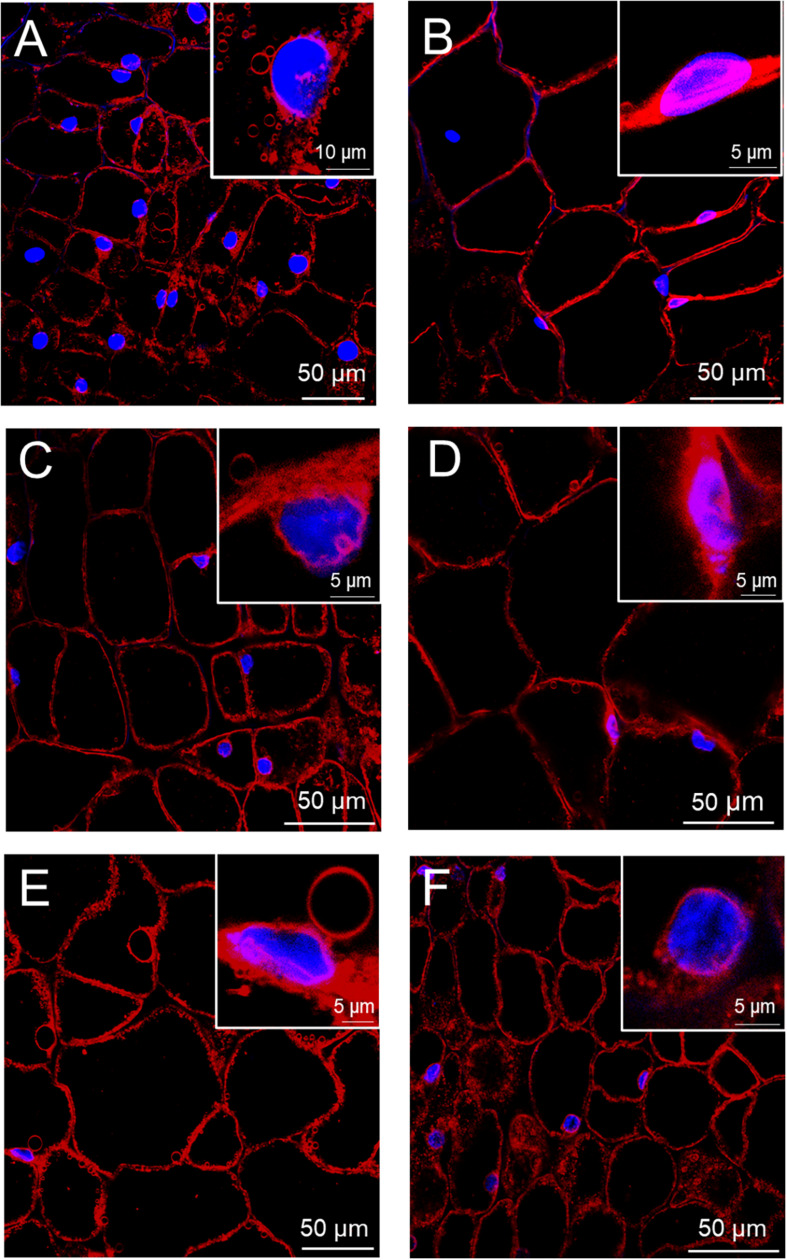
Progressive cell and nuclear damages in beetroots flooded for several days. Thick (∼1 mm) sections were taken from control beetroots **(A)** or from beetroots flooded for 1 day **(B)**, 2 days **(C)**, 3 days **(D)**, 4 days **(E)**, and 5 days **(F)**. Nuclei were stained with DAPI (blue) and membranes with SynaptoRed^TM^ C2 (red). Confocal microscope images were obtained with a Zeiss multiphoton microscope as described in the section “Materials and Methods.” Insets show magnified images of selected nuclei.

The observations of multiphoton microscopy allowed the recognition of morphological changes in beetroot cells during flooding. The cells of non-flooded roots showed the typical isodiametric shape of parenchyma cells with large central vacuoles and nuclei with near-spherical shapes. These nuclei mostly presented the homogeneously distributed chromatin that is characteristic in non-mitotic cells in mature tissues (Figure 2A). In contrast, after 1 day of flood stress, the cells showed an increase in size, probably due to the accumulation of water in vacuoles. This vacuole enlargement caused the confinement of cytoplasm and nuclei at the periphery of cells. In addition, chromatin condensation produced smaller nuclei (Figure 2B). On the second day of flood stress, the protoplasts of parenchyma cells were detached from cell walls while maintaining the isodiametric shape. Nuclei showed more irregular shapes with wrinkled membranes and fragmentation of chromatin (Figure 2C). On days 3 and 4 of flood stress, chromatin in nuclei appeared more compacted and fragmented (Figures 2D,E). Finally, on the fifth day of flooding, SynaptoRed-stained material was abundant in the cytoplasm, while nuclei showed degraded chromatin and ruptured membranes (Figure 2F and Supplementary Figure 1).

**FIGURE 3:**
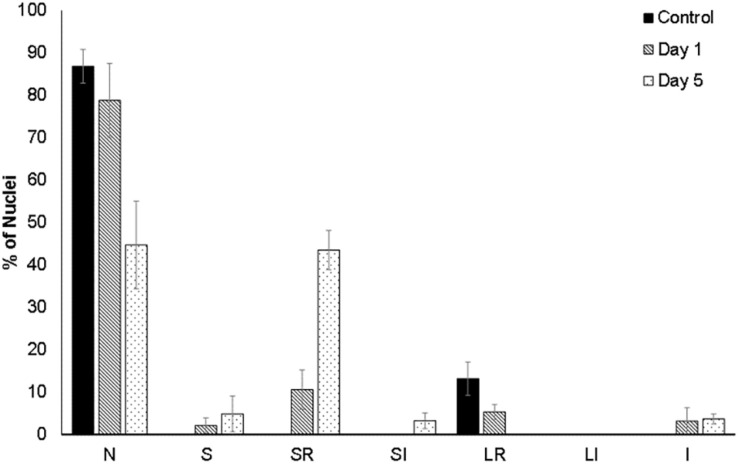
Morphometric analysis of the cell nuclei of beetroots. Using the ImageJ plug-in from the study by Filippi-Chiela et al. (2012), nuclei in cells from control (non-flooded) beetroots and from beetroots flooded for either 1 day or 5 days, were classified according to their size and irregularities as follows: N, normal nuclei; S, small (mitotic); SR, small and regular (apoptotic); SI, small and irregular; LR, large regular (senescent); LI, large irregular; I, irregular. At least, 60 nuclei (observed in several sections of one beetroot) were analyzed for each treatment.

For a quantitative assessment of nuclear alterations, a nuclear morphometric analysis was performed on DAPI-stained nuclei (Supplementary Figure 1), as described by Filippi-Chiela et al. (2012). This method classifies the nuclei according to their size and irregularities, allowing to spot senescent, mitotic, or apoptotic nuclei. This analysis showed that small-regular (SR) nuclei, considered to be apoptotic, were absent in non-flooded beetroots. In contrast, 12.6% of nuclei were SR after 1 day of flooding, and 43.9% in beetroots flooded for 5 days (Figure 3).

### Oxidative Phosphorylation and HK Activity Decreased in Mitochondria From Flood-Stressed Beetroots

**FIGURE 4:**
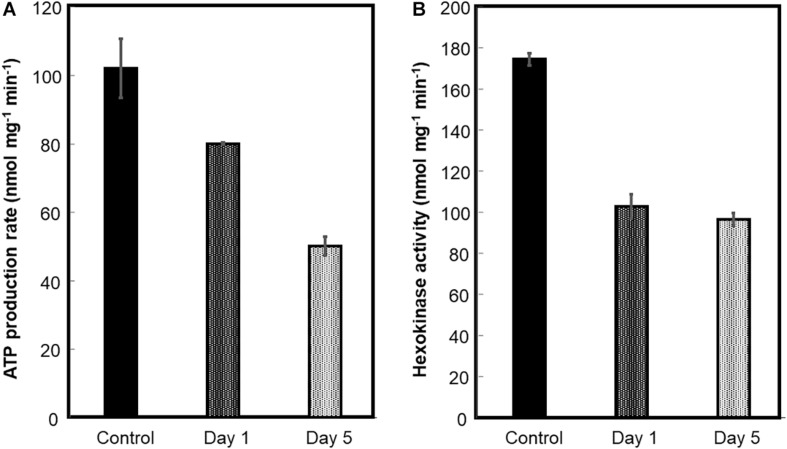
ATP production by oxidative phosphorylation and hexokinase (HK) activity of beetroot mitochondria. Mitochondria were prepared from control and flood-treated beetroots for 1 day or 5 days. The ATP production by oxidative phosphorylation **(A)** and HK activity **(B)** was measured as described in the section “Materials and Methods.” The data were represented as the mean ± SD of three experimental determinations.

Mitochondria were prepared from beetroots as indicated in the section “Materials and Methods.” To estimate the functional integrity of these mitochondria, the production rate of ATP by oxidative phosphorylation was measured in recently prepared mitochondria from control and flood-stressed plants. Compared with mitochondria from control beetroots, oxidative phosphorylation decreased ∼20% after 1 day of flood treatment. A larger decrease was observed in mitochondria from plants stressed for 5 days: the production rate of ATP was less than 50% of that observed in unstressed mitochondria (Figure 4A).

Hexokinase is located mainly on the OMM, functionally bound to VDAC (Alcántar-Aguirre et al., 2013). Hence, HK activity was measured to estimate the detachment of this enzyme from OMM, or the loss of this membrane, caused by flood stress. HK-specific activity decreased by ∼42% on the first day of flood-stress treatment and by ∼45% on the fifth day (Figure 4B).

### Cytochrome c Was Released From Mitochondria During Flood Stress

**FIGURE 5:**
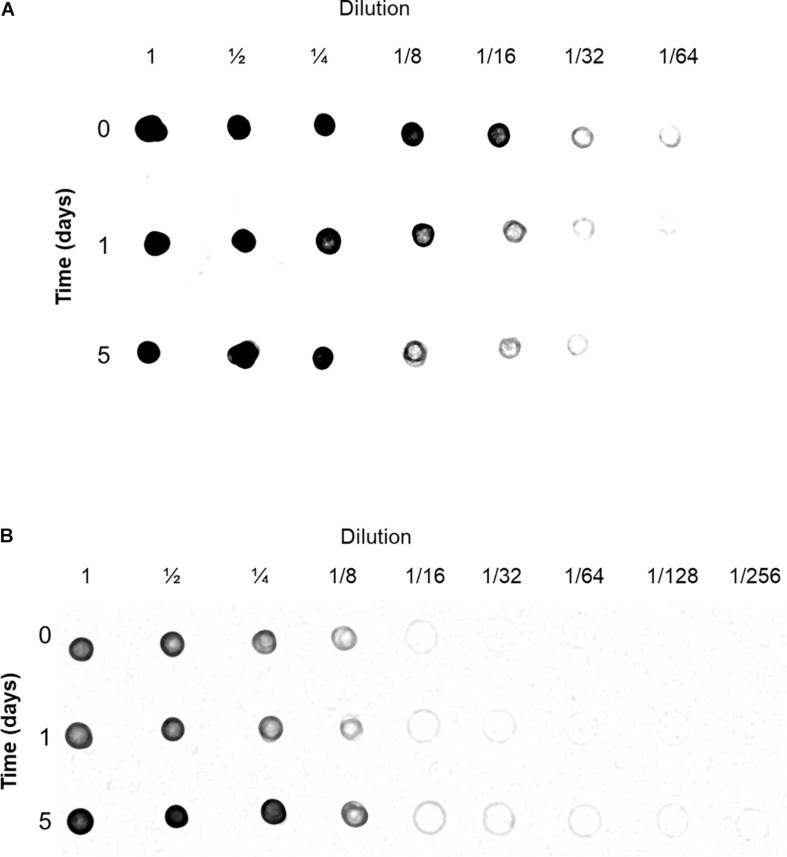
Detection of cytochrome c (cyt c) in mitochondria **(A)** and cytoplasm **(B)** fractions. Samples from control and flood-treated beetroots (for 1 day or 5 days) with known amounts of protein were serially diluted with Tris-buffered saline (TBS). Aliquots of 5 μl were blotted onto a membrane of nitrocellulose, dried, and immunodetected with anti-*Arabidopsis* thaliana cyt c antibodies. This experiment was performed three times; a representative immunoblot image is shown in this figure.

During the preparation of mitochondria, the 21,000 × *g* supernatant was kept and considered to contain cytoplasmic solutes. The cyt c content of both mitochondria and cytoplasm from control and flood-treated beetroots were estimated by immunodetection (Dot blot) with an anti-*Arabidopsis* cyt c antibody. This cytochrome was found in both mitochondria and cytoplasm. The greatest amount of cyt c in the mitochondrial fraction was found in the control sample (Figure 5A, time 0). This contrasts with the cytoplasmic fraction, where the highest amount of cyt c was found in beetroots flood-treated for 5 days (Figure 5B, time 5), where this antigen was observed even in the 1:256 dilution. These results indicate that, most probably, cyt c was released from mitochondria during the flood-stress treatments applied.

### Voltage-Dependent Anion-Selective Channel Abundance Was Higher in Mitochondria From Beetroots Flood-Stressed for 1 Day

**FIGURE 6:**
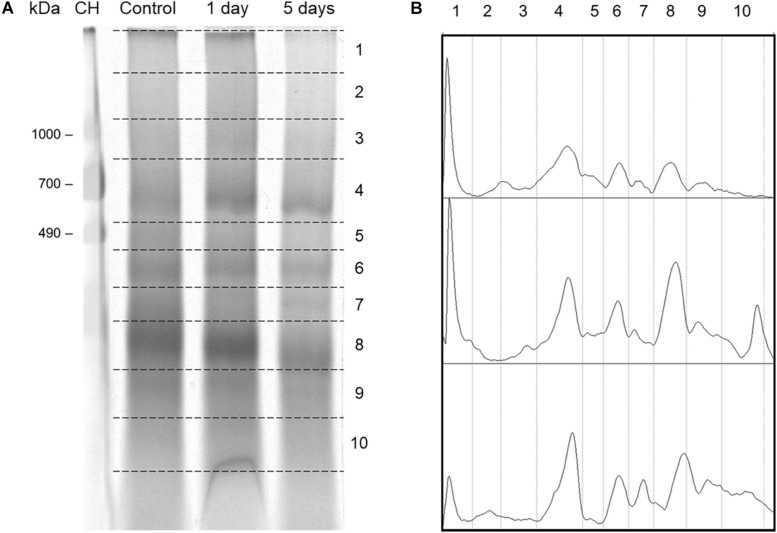
Separation of mitochondrial protein complexes from control and flood-stressed beetroots by Blue Native PAGE (BN-PAGE) for the identification of their proteins by mass spectrometry. **(A)** A stained BN-PAGE gel image. Mitochondrial protein complexes from control and flood-stressed beetroots for 1 day or 5 days were run in a 4–13% polyacrylamide gradient gel. Broken lines limit the sections cut for its analysis by mass spectrometry. In the first lane, chicken heart homogenate with 50 μg of protein was run as molecular mass markers. Identified mitochondrial proteins are listed in Supplementary Table 1. **(B)** Densitometric traces of the gel image in **(A)** were obtained using ImageJ. Calculated mean molecular masses (in kDa) in each gel numbered section were as follows: 2, 1,378; 3, 974; 4, 653; 5, 474; 6, 363; 7, 286; 8, 202; 9, 135; and 10, 93. This experiment was replicated three times (one of them omitting formaldehyde during the preparation of mitochondria), obtaining very similar results.

To observe the mitochondrial protein complexes in control and flood-stressed beetroots, mitochondria were treated with the detergent DDM at a detergent/protein ratio of 2 (a ratio previously determined to be optimal). Solubilized protein complexes were then separated by Blue Native electrophoresis (Wittig et al., 2006). After staining the gel, the densitometric traces in Figure 6B were obtained. In them, an increase in the intensity of stained protein bands was observed in sections 4–8 (corresponding to molecular masses from 700 to 200 kDa) in response to flood treatment. Later, a lane from each day of treatment was sliced into 10 sections (Figure 6A), and proteins in each section were identified by tandem mass spectrometry (MS/MS).

A total of 76 mitochondrial proteins were identified by MS/MS (Supplementary Table 1). Protein complexes producing most peptide fragmentation spectra were the ATP synthase (Complex V), NADH dehydrogenase (Complex I), and ubiquinol-cytochrome c oxidoreductase (Complex III). Other abundant proteins (as estimated by their explained spectra count) were the mitochondrial transporters for ADP/ATP, phosphate, and dicarboxylates/tricarboxylates. Chaperonins CPN60-2 and CPN60-like 2 presented ∼100 explained fragmentation spectra in all conditions.

**FIGURE 7:**
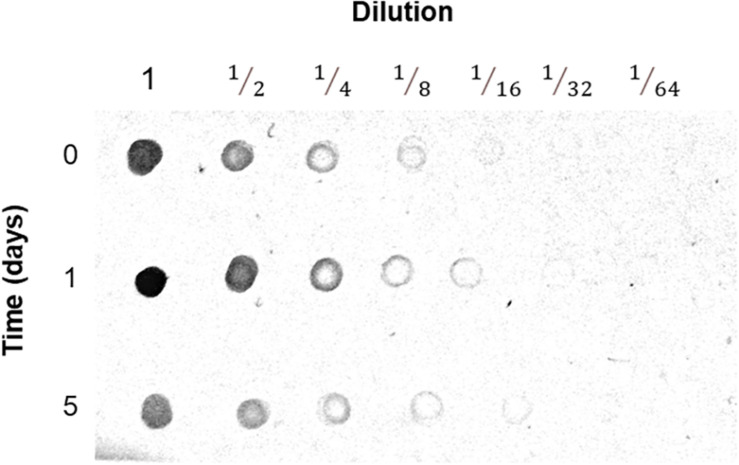
Detection of the voltage-dependent anion-selective channel (VDAC) in mitochondria. Mitochondria prepared from control and flood-treated beetroots (for 1 day or 5 days) with known amounts of protein were serially diluted with TBS. Aliquots of 5 μl were blotted onto a nitrocellulose membrane, dried, and immunodetected with anti-VDAC antibodies. In this figure, the results of one of the three experiments performed are presented.

Few proteins produced more peptide spectra when extracted from flood-stressed beetroots. Most noticeably, VDACs produced 2.7 times more peptides after 1 day of flood treatment. After 5 days of flood treatment, VDAC spectra counts returned to 83% of control levels (Supplementary Table 1). These changes in VDAC abundance were confirmed immunologically: in Figure 7, the results of a Dot blot experiment are presented. In this study, in almost all dilutions, it is observed that the abundance of immunodetected VDAC was higher in mitochondria from beetroots subjected to flooding for 1 day. After 5 days, VDAC content was lower than that found in control mitochondria. The densitometric analysis of the Dot blot in Figure 7 allowed us to estimate that VDAC content in mitochondria increased 1.9 times when beetroots were flood-stressed for 1 day and decreased to 46% of control values in beetroots flooded for 5 days (Supplementary Table 2). These results are consistent with those estimated by spectra counting, as shown in Supplementary Table 1.

Not all OMM proteins behaved the same way similar to VDAC. Spectra counts of HK, for instance, decreased by 87% and 58% of control values in mitochondria from beetroots flooded for 1 day and 5 days, respectively. These values are comparable with the activity of HK measured in isolated mitochondria (Figure 4B).

### Protein Complexes Formed by VDAC Differed in Size in Mitochondria From Control and Flood-Stressed Beetroots

In addition to their changes in abundance, VDAC proteins showed changes in the size of the complexes they form in beetroots subjected to flooding stress. In mitochondria from control beetroots, three VDAC isoforms mainly form complexes with masses of ∼93 kDa (VDAC-2 and 34 kDa VDAC-like) or ∼135 kDa (VDAC of 36 kDa). However, in mitochondria from beetroots flood-stressed for 1 day, these three VDAC isoforms appeared mainly in complexes with a mass of ∼202 kDa, indicating a possible formation of hexamers or complexes with other proteins. On the fifth day of flooding treatment, VDAC proteins reverted to form complexes with lower masses, i.e., ∼93–135 kDa (Supplementary Table 3).

For analyzing the possible associations between mitochondrial proteins, the tool NOVA was used (Giese et al., 2015). With it, the migration profiles of identified proteins were used to perform the hierarchical clustering shown in Supplementary Figure 2. This clustering analysis grouped well the proteins in respiratory chain complexes. For instance, proteins of Complex III were grouped in a ∼363 kDa complex in mitochondria from both control and flood-treated beetroots. Other complexes are formed by prohibitin: two isoforms of it were found migrating at more than 1,300 kDa, without presenting significant changes with the flood treatments.

**FIGURE 8:**
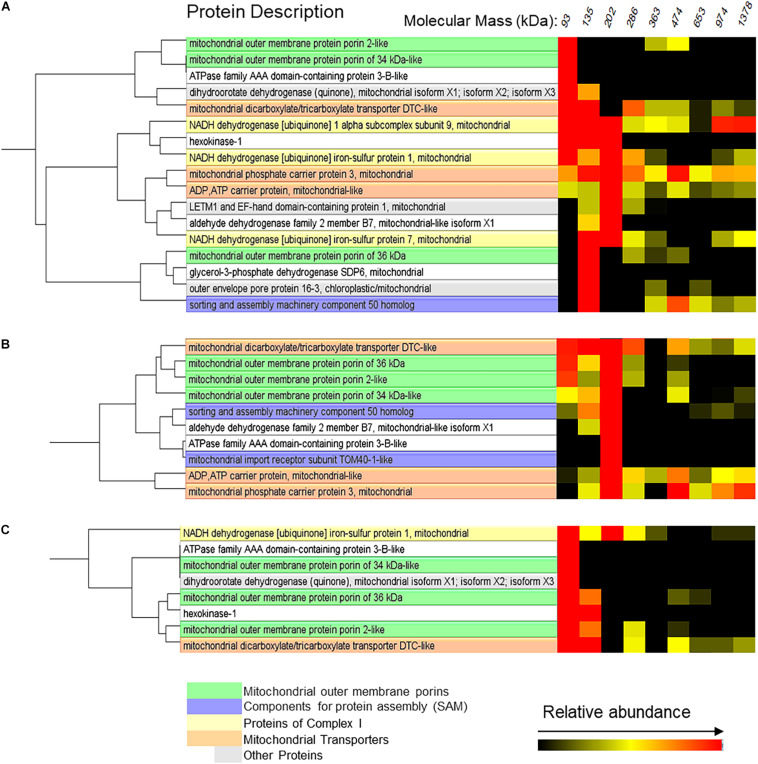
Mitochondrial proteins probably interacting with VDAC in control beetroots **(A)** or beetroots flooded for 1 day **(B)** or 5 days **(C)**. The cluster analysis of the proteins separated by the BN-PAGE in Figure 6 was performed using the software NOVA. Only the proteins in complexes with a mass distribution similar to that formed by VDAC are shown in this figure. The distribution profiles of all identified mitochondrial proteins are in Supplementary Figure 2.

The possible interactions of VDAC with other proteins are illustrated in Figure 8. Two proteins appeared forming complexes of the same mass as those formed by VDAC in mitochondria from both control and flood-treated beetroots: the ATPase family AAA domain-containing protein 3B-like and the mitochondrial dicarboxylate/tricarboxylate transporter DTC-like (DTCL). In mitochondria from control beetroots, VDAC-2 and 34 kDa VDAC-like appeared to be associated together and with dihydroorotate dehydrogenase, in addition to the abovementioned proteins. The 36 kDa VDAC is associated mainly with the outer envelope pore protein 16-3 and with the glycerol-3-phosphate dehydrogenase SDP6 (Figure 8A). After 1 day of flood stress, the three VDAC isoforms clustered together and with DTCL (Figure 8B). Finally, after the 5-day flood-stress treatment, VDAC (especially 36 kDa VDAC) was found to be associated with HK in small (∼93 to ∼135 kDa) complexes (Figure 8C).

Other proteins presented changes in their complex-size distribution. One was chaperonin CPN60-2, which formed high molecular mass complexes (mainly of ∼653 kDa) under control conditions (Supplementary Figure 2A), while it formed mostly ∼286 kDa complexes under flood stress (Supplementary Figures 2B,C). The ADP/ATP carrier protein (XP_010689049.1) is another protein that presented changes in its distribution: it formed medium-sized complexes (mostly of ∼286 kDa) in well-drained beetroots and much heavier (>1,300 kDa) complexes in beetroots flooded for 1 day. Finally, the mitochondrial import receptor subunit TOM40-1-like formed complexes of ∼202 and ∼474 kDa. These heavier complexes were mostly found in control conditions, while ∼202 kDa complexes were enriched in flood-stressed beetroots (Supplementary Figure 2).

## Discussion

Beetroot cells are sensitive to anoxia and dependent on mitochondrial respiration for their energy needs (Petraglia and Poole, 1980). The anoxia caused by flooding inhibits growth and produces PCD (Chen et al., 2014; Kamal and Komatsu, 2015; Qi et al., 2018). In the roots of some plant species, the flood-induced PCD causes the formation of aerenchyma (Ni et al., 2019). This tissue, formed by air-filled dead cortex cells, enhances the aeration of root cells, facilitating the adaption of the plant to the hypoxia produced by flooding (Evans, 2003; Voesenek and Bailey-Serres, 2015). To our knowledge, there are no reports of aerenchyma formation in flooded beetroots.

Some structural changes in cellular and subcellular architecture such as chromatin condensation, cell shrinkage, nuclear deformation, and, in mammalian cells, the formation of apoptotic bodies and its subsequent degradation by phagocytosis are considered diagnostic signs of PCD (Lord and Gunawardena, 2012; Dickman et al., 2017). In this study, the nuclear morphological changes were observed in control and flood-stressed beetroot cells by multiphoton fluorescence microscopy. We observed that the nuclei presented a decrease in size and chromatin condensation in cells from beetroots flood-treated for 1 day, while in samples treated for 5 days, many nuclei were completely fragmented (Figure 2). According to the index of nuclear irregularity (Filippi-Chiela et al., 2012), no nuclei deemed as apoptotic were observed in control beetroots. In contrast, almost half of the nuclei analyzed were classified as apoptotic in flood-stressed beetroots (Figure 3).

Another common feature in PCD is the release of cyt c from mitochondria to the cytoplasm, due to the permeabilization of OMM. This release of cyt c has been reported in a variety of organisms, such as yeast, mammals, and plants (Balk et al., 1999; Sun et al., 1999; Martínez-Fábregas et al., 2013), although its role in plant PCD is still under debate (Zhang et al., 2020). In this study, cyt c was detected in both the mitochondrial and cytosol fractions in control and flood-treated beetroots. However, cyt c content increased in the cytosol after 1 day of flooding. This change was accompanied by a decrease in the amount of cyt c in mitochondria, which suggested cyt c leaked from mitochondria to the cytoplasm. These results, together with the observed nuclear fragmentation, indicated that flooding is an appropriate method to induce PCD in beetroots.

Mitochondria play important roles in responses to stress and plant survival during oxidative stress. When plant mitochondria receive PCD-inducing signals (mainly ROS and Ca^2+^ ions), the MPTP opens (Van Aken and Van Breusegem, 2015). There is no consensus on the protein components that constitute MPTP. On the one hand, it has been proposed to be formed mainly by VDAC probably bound to inner membrane proteins such as the ADP/ATP carrier or the phosphate mitochondrial translocator (Takahashi and Tateda, 2013; Hurst et al., 2017). On the other hand, it has been proposed that the dimerization of the ATP synthase forms an ion channel through its intramembrane domain (Bernardi et al., 2015).

The BN-PAGE was used in this study to separate protein complexes (Wittig et al., 2006), identifying the proteins in each section of these gels by mass spectrometry (Senkler et al., 2017). This method allowed us to estimate the abundance (by spectra counting) of each identified protein and to detect changes in the masses and the possible composition of mitochondrial protein complexes. Among the proteins that show changes in response to flooding are VDAC isoforms. These OMM channels increase in mitochondria from flooded beetroots (Supplementary Table 1 and Figure 7). This increase has been observed in soybean in response to flooding stress (Komatsu et al., 2011), in elm seeds when PCD was induced by a ROS increase during aging (Li et al., 2017), and in PCD induced by ceramide in rice (Zhang et al., 2020). In *Arabidopsis*, an increase in the VDAC protein Hsr2 was observed after heat- and senescence-induced PCD (Swidzinski et al., 2004). In addition, protein complexes formed by VDAC changed in size with flood stress. In mitochondria from control beetroots, they were found forming mainly trimers or tetramers. However, in mitochondria from beetroots flood-stressed for 1 day, VDAC formed complexes with a mass of ∼202 kDa, suggestive of hexamers. These different VDAC oligomers have been observed in *Arabidopsis* (Klodmann et al., 2011; Senkler et al., 2017) and potato OMM (Hoogenboom et al., 2007). The changes in VDAC oligomerization have been associated with the permeabilization of OMM that causes cyt c release in both animal (Zalk et al., 2005; Shoshan-Barmatz et al., 2008, 2010) and plant cells (reviewed in Takahashi and Tateda, 2013).

In addition to forming oligomers of different sizes, VDAC could associate with other proteins. These associations could change in the process of flood-induced PCD. Among the mitochondrial transporters, the DTCL appeared to form complexes of similar sizes than those formed by VDAC in mitochondria from both control and flood-stressed beetroots. A possible association of DTCL with VDAC has been observed in *Arabidopsis* (Klodmann et al., 2011). DTCL was induced in the halophyte *Suaeda asparagoides* under salt stress (Ayarpadikannan et al., 2012), but its possible role in flood-induced PCD is yet to be studied. The ADP/ATP carrier and the mitochondrial phosphate carrier 3, which have been proposed to be the components of different mitochondrial membrane complexes such as the ATP synthase (Pedersen, 2008) and the MPTP (Takahashi and Tateda, 2013), form, accordingly, complexes with very diverse masses.

Hexokinase associates with VDAC, inhibiting apoptosis by preventing the release of cyt c in mammalian mitochondria (Azoulay-Zohar et al., 2004; Abu-Hamad et al., 2008; Shoshan-Barmatz et al., 2008). There is also evidence indicating that HK bound to VDAC inhibits the formation of MPTP in plant mitochondria (Godbole et al., 2013; Rodríguez Saavedra et al., 2021). A functional association between HK and VDAC has been reported in beetroots, where it was observed that the ATP produced by oxidative phosphorylation in the mitochondrial matrix is preferentially channeled toward the HK bound to VDAC (Alcántar-Aguirre et al., 2013). In this study, both the activity and the peptide spectra produced by HK decreased in response to flooding stress. HK appeared, in all treatments, in small (∼140 kDa) complexes. This mass is consistent with that of an HK molecule bound to a VDAC trimer.

Finally, the mitochondrial import receptor TOM-40 and the chaperonin CPN60-2 presented changes in the masses of the complexes they form in flood-stressed beetroots. CPN60-2 increased and formed smaller complexes in flood-stressed beetroots. CPN60-2 protein increased in flood-stressed soybean (Komatsu et al., 2011). Similarly, the mitochondrial chaperonin Hsp60 increased (and was released from mitochondria) after a heat shock in *Arabidopsis* (Rikhvanov et al., 2007).

## Conclusion

The results of this study indicated that flooding stress is a good method to induce PCD in beetroots. Using it, it was observed that VDAC, and possibly other proteins, appeared to be involved in the process of PCD induced by flooding stress.

## Data Availability Statement

The data presented in the study are deposited in the ProteomeXchange Consortium *via* the PRIDE partner repository under accession number: PXD027781.

## Author Contributions

KR-M and LG designed this study and wrote the manuscript. KR-M performed most of the research. BL provided technical assistance and advice throughout this study. LS performed the microscopy work. AC provided valuable advice and analyzed proteins by MS. All authors contributed to the article and approved the submitted version.

## Conflict of Interest

The authors declare that the research was conducted in the absence of any commercial or financial relationships that could be construed as a potential conflict of interest.

## Publisher’s Note

All claims expressed in this article are solely those of the authors and do not necessarily represent those of their affiliated organizations, or those of the publisher, the editors and the reviewers. Any product that may be evaluated in this article, or claim that may be made by its manufacturer, is not guaranteed or endorsed by the publisher.
