# Supplementary figures and images for: Voltage-Dependent Anion-Selective Channels and Other Mitochondrial Membrane Proteins Form Diverse Complexes in Beetroots Subjected to Flood-Induced Programmed Cell Death
Source: Front Plant Sci. 2021 Sep 8;12:714847. doi: 10.3389/fpls.2021.714847 (PMC8457146; doi:10.3389/fpls.2021.714847)

## Slide 1
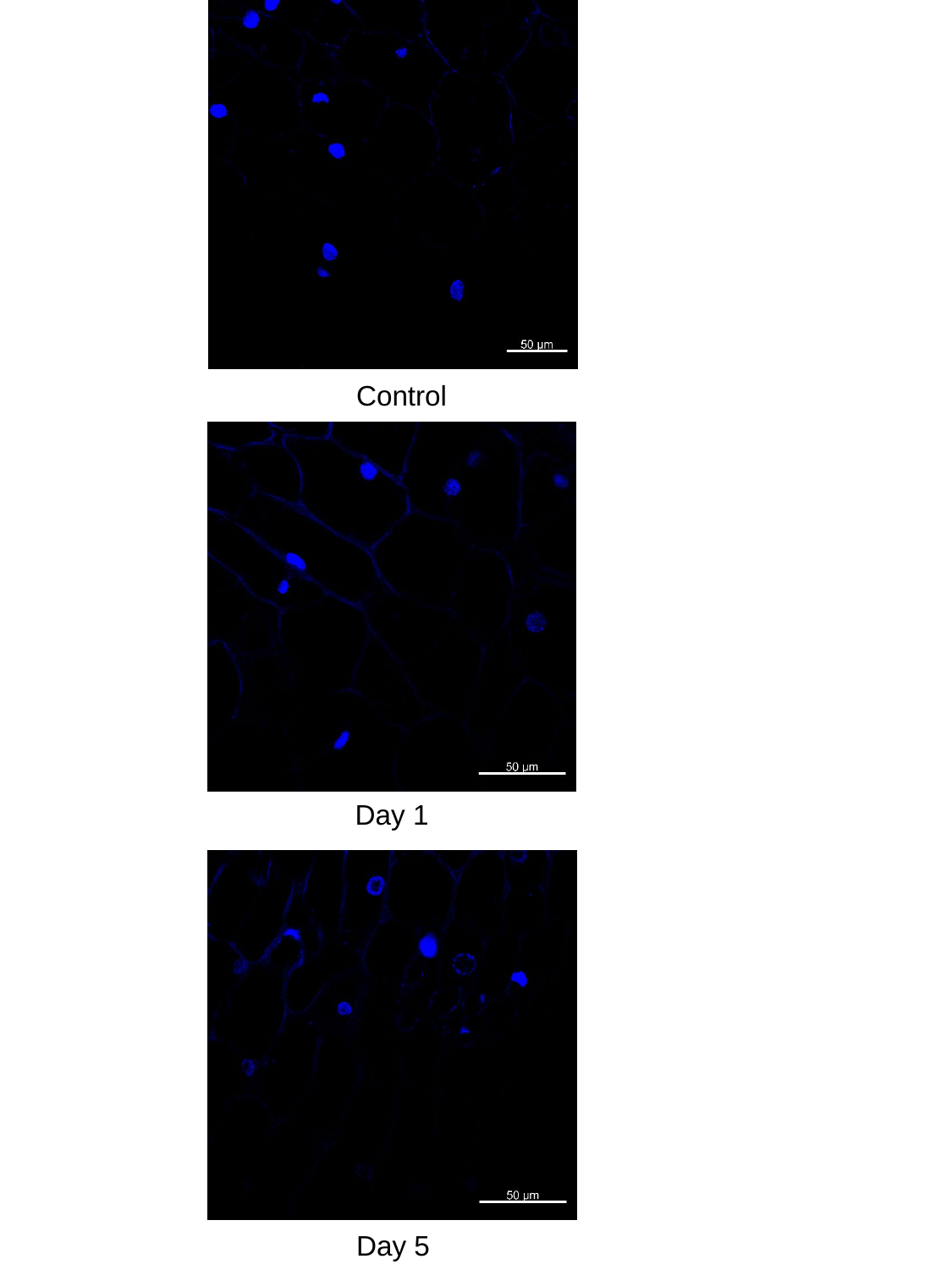

Control
Day 1
Day 5

Supplement: Supplementary file 1 [file Presentation_1.PPTX]
