# Supplementary material for: Voltage-Dependent Anion-Selective Channels and Other Mitochondrial Membrane Proteins Form Diverse Complexes in Beetroots Subjected to Flood-Induced Programmed Cell Death
Source: Front Plant Sci. 2021 Sep 8;12:714847. doi: 10.3389/fpls.2021.714847 (PMC8457146; doi:10.3389/fpls.2021.714847)
Supplement: Supplementary file 2 [file Presentation_2.PPTX]

## Slide 1
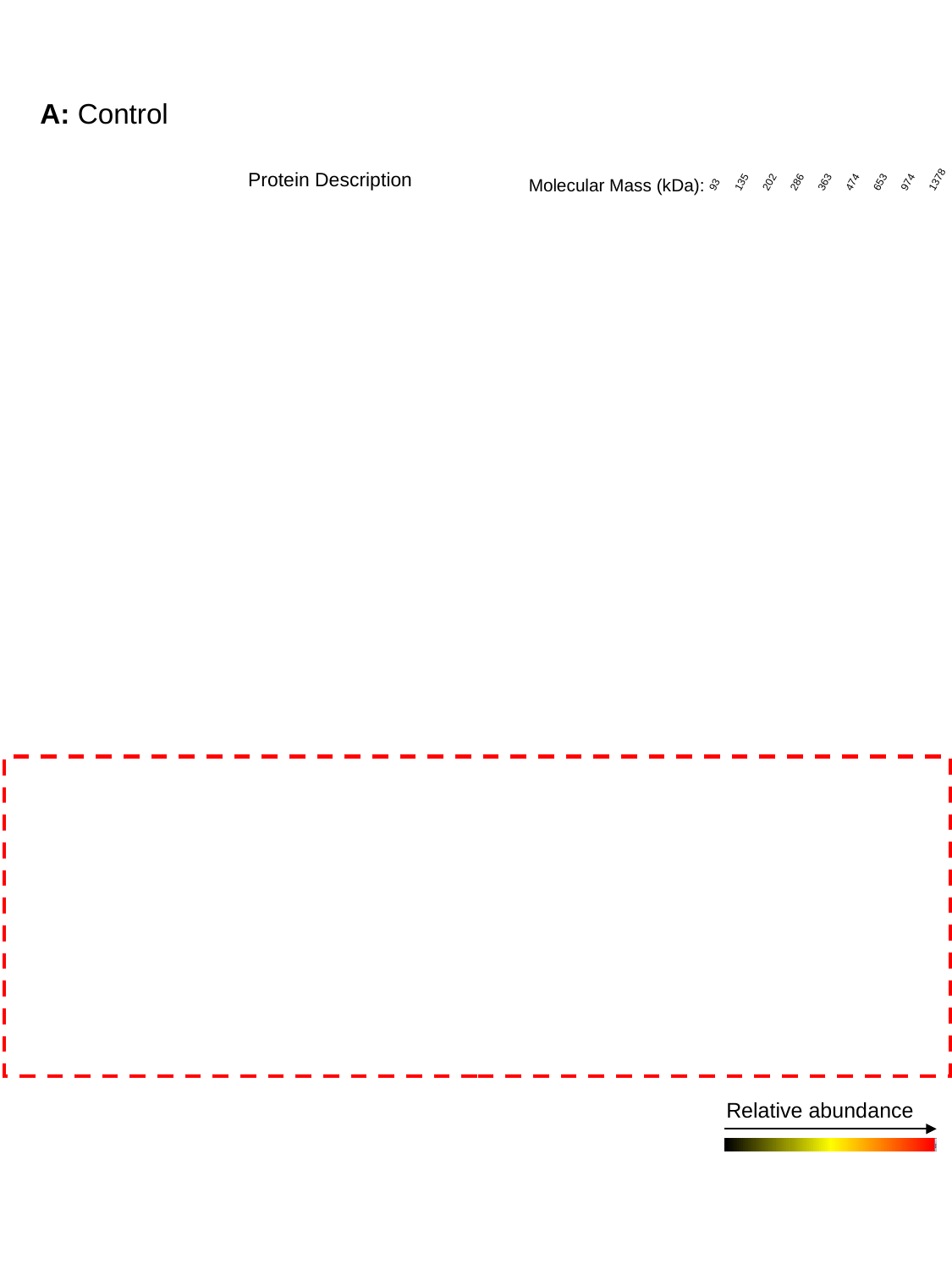

A: Control
Protein Description
Molecular Mass (kDa):
Relative abundance

## Slide 2
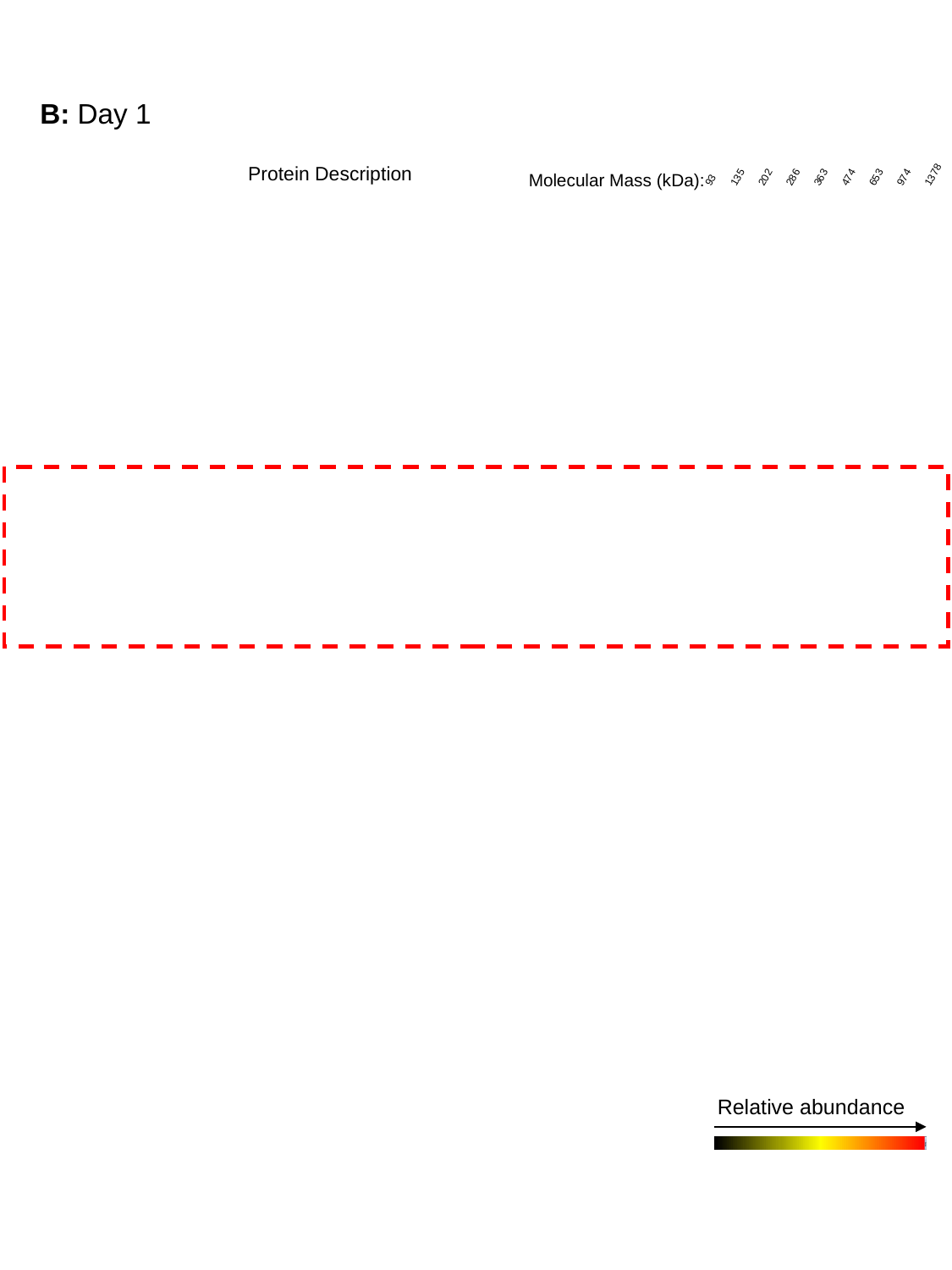

B: Day 1
Protein Description
Molecular Mass (kDa):
Relative abundance

## Slide 3
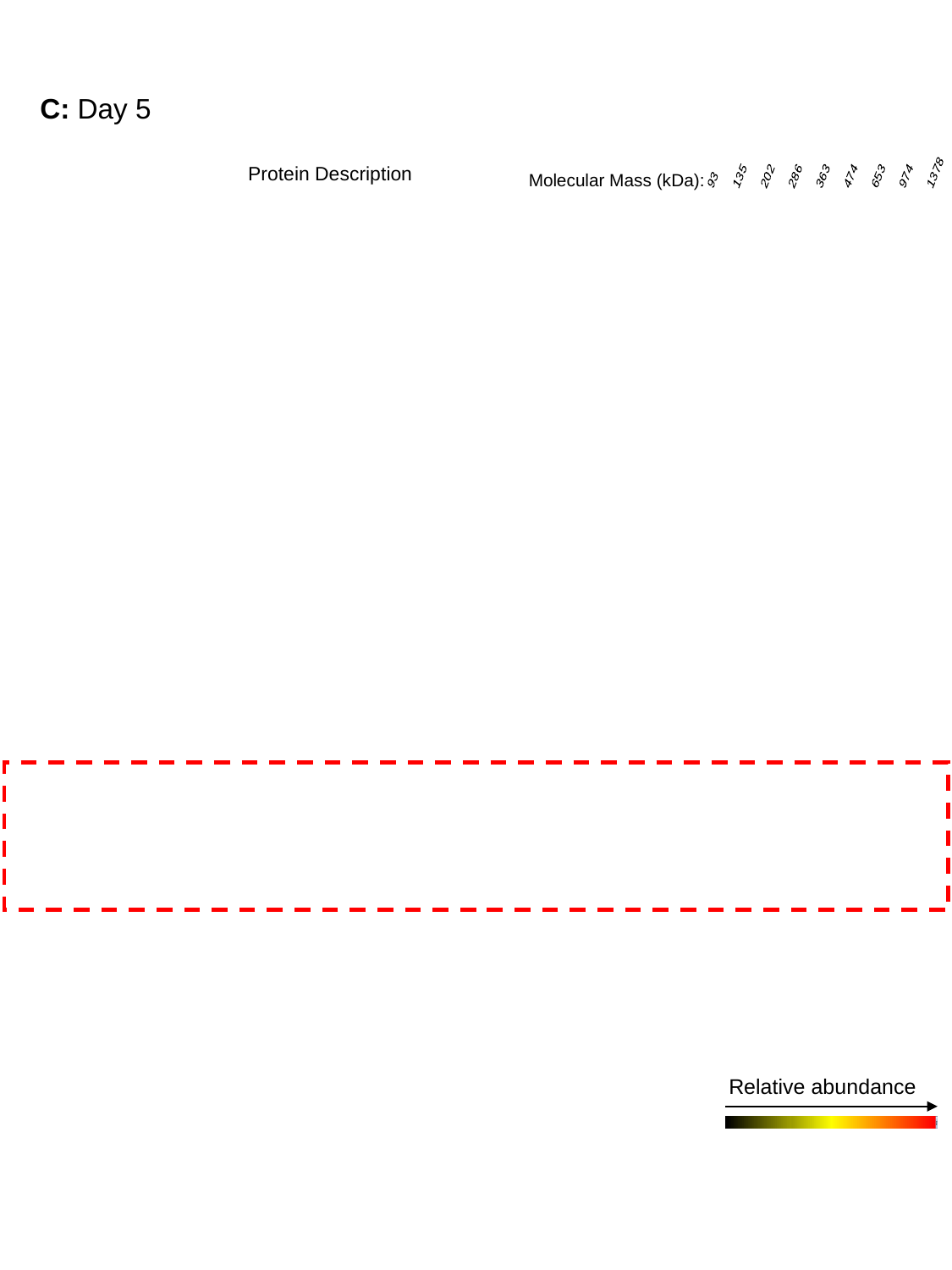

C: Day 5
Protein Description
Molecular Mass (kDa):
Relative abundance
